# A retrospective cohort study of incidence and risk factors for severe SARS-CoV-2 breakthrough infection among fully vaccinated people

**DOI:** 10.1038/s41598-023-35591-w

**Published:** 2023-05-26

**Authors:** Tatjana Meister, Anastassia Kolde, Krista Fischer, Heti Pisarev, Raivo Kolde, Ruth Kalda, Kadri Suija, Anna Tisler, Anneli Uusküla

**Affiliations:** 1 Institute of Family Medicine and Public Health, Faculty of Medicine, University of Tartu, Tartu, Estonia; 2 Institute of Mathematics and Statistics, Faculty of Science and Technology, University of Tartu, Tartu, Estonia; 3 Institute of Genomics, Faculty of Science and Technology, University of Tartu, Tartu, Estonia; 4 Institute of Computer Science, Faculty of Science and Technology, University of Tartu, Tartu, Estonia

**Keywords:** Diseases, Risk factors

## Abstract

SARS-CoV-2 vaccination is currently the mainstay in combating the COVID-19 pandemic. However, there are still people among vaccinated individuals suffering from severe forms of the disease. We conducted a retrospective cohort study based on data from nationwide e-health databases. The study included 184,132 individuals who were SARS-CoV-2 infection-naive and had received at least a primary series of COVID-19 vaccination. The incidence of BTI (*breakthrough infection*) was 8.03 (95% CI [*confidence interval*] 7.95⎼8.13/10,000 person-days), and for severe COVID-19 it was 0.093 (95% CI 0.084⎼ 0.104/10,000 person-days). The protective effect of vaccination against severe COVID-19 remained constant for up to six months, and the booster dose offered an additional pronounced benefit (hospitalization aHR *0.32, 95% CI 0.19⎼0.54).* The risk of severe COVID-19 was higher among those ≥ 50 years of age (aHR [*adjusted hazard ratio*] 2.06, 95% CI 1.25⎼3.42) and increased constantly with every decade of life. Male sex (aHR 1.32, 95% CI 1.16⎼1.45), CCI (*The Charlson Comorbidity Index*) score ≥ 1 (aHR 2.09, 95% CI 1.54⎼2.83), and a range of comorbidities were associated with an increased risk of COVID-19 hospitalization. There are identifiable subgroups of COVID-19-vaccinated individuals at high risk of hospitalization due to SARS-CoV-2 infection. This information is crucial to driving vaccination programs and planning treatment strategies**.**

## Introduction

Large-scale vaccination programs against severe acute respiratory syndrome coronavirus 2 (SARS-CoV-2) deployed by governments and health authorities in more than 200 countries^1^ have halted coronavirus disease 2019 (COVID-19)-associated mortality and caused people worldwide to anticipate the oncoming end of the pandemic.

Clinical trials, as well as studies tracking real-world data, demonstrated the pronounced effectiveness of COVID-19 vaccines against SARS-CoV-2 infection and severe COVID-19^2^.

People at increased risk of severe COVID-19 were among the first to be involved in vaccination programs in many countries^3,4^. This probably helped prevent the collapse of health systems in these countries and maintain economic activity^5^. By the end of the second year of the pandemic, the increased number of people with vaccine-induced immunity together with the emergence of new, more contagious but less virulent strains of the virus yielded a drop in COVID-19-associated hospitalizations and deaths worldwide^6^. These developments reduced caution regarding COVID-19 and led to alleviation of coronavirus-related containments. Therefore, the "flattening the curve" strategy with a main emphasis on limiting population mobility and contact tracing was replaced by less stringent measures and focused on the protection of vulnerable populations by encouraging vaccine uptake^7,8^.

Vaccines prevent severe COVID-19, although their ability to prevent infections even in highly vaccinated populations fades due to the ability of new strains to evade the immune response^9–12^. Vaccinated individuals account for at least one-third of all people hospitalized due to COVID-19. Thus, the threat of COVID-19 is not over^13–16^. Evidence is needed to target people who are at risk of severe infection with timely boosters, antiviral treatment, and preexposure prevention.

A great deal of uncertainty exists in terms of future planning of vaccination strategies alongside conflicting results in studies aimed at exploring the long-term protection gained by vaccination or booster shot regimens^17–20^. We aimed to evaluate the risk of breakthrough infections focusing on severe COVID-19 requiring hospital care and associated sociodemographic and health-related factors.

## Methods

### Design

We performed a retrospective cohort study based on individual-level data linking national routine datasets on laboratory-confirmed SARS-CoV-2 infection, COVID-19 vaccination status, and health care utilization between 19 January 2021 and 9 February 2022 in a SARS-CoV-2 infection-naive fully vaccinated cohort of 184,132 individuals. This study follows STROBE guidelines for observational research reporting^21^.

All research was performed in accordance with relevant guidelines and regulations. The Research Ethics Committee of the University of Tartu approved the study by the 18th of October 2021(No. 351/M-8). The need for informed consent was waived by the Ethics committee of the University of Tartu due to the retrospective nature of the study.

The funding agencies had no role in the study design, data collection, data analysis, interpretation, or writing of the manuscript.

### Study population

Individuals aged 12 years and older not previously infected with SARS-CoV-2 who had a primary vaccine series against SARS-CoV-2 with at least two doses of BNT162b2 (Pfizer/BioNTech), mRNA-1273 (Moderna), AZD1222 (Oxford/AstraZeneca) or one dose of Ad26.COV2. (Janssen/Johnson & Johnson) vaccine were included. Individuals who had a supplementary dose of vaccine after the primary series were considered boosted. Data are presented in the supplementary materials.

A detailed description of study cohort and participants' numbers in each group is presented in flow diagram (see Appendix A).

### Study period

The study period was from 19 January 2021 to 9 February 2022. The data were pertinent to the first two years of the COVID-19 pandemic (driven by the alpha, delta, and, to a lesser extent, the omicron variants of SARS-CoV-2)^22^. This period was characterized by free access to SARS-CoV-2 PCR testing and subsequent treatment for all Estonian citizens suspected to have or who had confirmed SARS-CoV-2 infection. By the end of the period, 65% of the Estonian population was fully vaccinated^23^.

### Data sources

#### SARS-CoV-2 vaccination and testing data

Data on COVID-19 vaccination (dates, vaccine, and the manufacturer for each vaccination episode) and SARS-CoV-2 testing dates (results, dates) were retrieved from the Health and Welfare Information Systems Centre (TEHIK)^24^. The TEHIK maintains the e-health system in Estonia (electronic health records, EHRs) by collecting records about all contacts with health care providers, including medical care and laboratory testing records, referrals, and data on vaccinations^25^. According to law, all health care providers and laboratories in Estonia are obliged to report to the TEHIK, with an expected 100% coverage. Cohort membership was defined based on the data from the TEHIK.

#### Health status and health care utilization data.

The Estonian Health Insurance Fund (EHIF) maintains records of all health care services delivered to all residents with valid health insurance (approximately 95% of all residents), including personal information (sex, age), healthcare-related services delivered to individuals, primary and other diagnoses on health care claims (based on the International Classification of Diseases, 10th revision (ICD-10)), treatment type (in- or outpatient), types of tests or services provided), and the date of death^26^.

#### Sociodemographic data

*The Population Register* is a unified database of Estonian citizens and foreign nationals living in Estonia on the basis of right of residence or residence permit and is managed and developed by the Ministry of the Interior. Population Register data were used to identify the study subjects’ location of residence (including emigration status), education, and ethnicity.

### Exposures

We considered a primary series of COVID-19 vaccine and boosters as exposure in this study.

COVID-19 primary vaccine series was defined as vaccine administered as the first dose for 1-dose series and the second dose for 2-dose series. Vaccine booster was defined as any additional SARS-CoV-2 vaccine dose received after completing of primary vaccination series against COVID-19.

### Outcomes

#### Breakthrough infection

Breakthrough infection (BTI) was defined as a SARS-CoV-2-positive laboratory test (PCR or antigen test) occurring at least 14 days after the last vaccination and was our main outcome. The time of breakthrough infection was based on the date of the positive test collection.

#### Severe BTI

A secondary outcome, severe BTI, was hospitalization related to COVID-19 three days before to 30 days after a laboratory-confirmed SARS-CoV-2 infection. We set additional criteria for COVID-19-related hospitalization, aiming to avoid misclassification related to COVID-19 cases identified by routine screening during hospitalization for other reasons. There had to be at least one of the following diagnoses in relation to hospitalization: COVID-19 (U07.1, U07.2), acute respiratory tract infections (J00– J06, J12, J15–J18, J20–J22, J46) or lower respiratory tract infections (J80–84, J85–J86)^27,28^.

### Covariates

We included possible confounders (covariates) associated with the severity of COVID-19 or associated with both SARS-CoV-2 infection and hospitalization.

#### Sociodemographic characteristics

The age, sex and education level of the study participants were included in the analysis.

The educational level of study participants derived from the population statistics was divided into three groups according to the International Standard Classification of Education (ISCED): low education level (basic education or below), medium education level (general secondary education or vocational education based on secondary education), and high education level (higher or tertiary education)^29,30^.

#### Prevaccination comorbidities

Based on health care claims data for the period of 24 months prior to vaccination, the *Charlson Comorbidity Index* (CCI) score was estimated. The CCI is a weighted index that accounts for the number of patient comorbidities and severity and has good predictive validity for the mortality of patients with COVID-19^31^. Comorbidity burden was categorized into three groups (CCI score = 0, CCI score 1–2, and CCI score ≥ 3) and calculated using algorithms and weights described by Quan et al.^32^.

We also used the duration (measured in weeks) of hospitalization 365 days pre-vaccination as a measure of the morbidity of the study participants^33^.

Data on selected chronic comorbid conditions were included (defined based on ICD-10 classification diagnosis codes), considering the potential of these diseases to act as risk factors for severe COVID-19 (see Appendix B). Comorbidities were defined as any secondary or other diagnoses coded in the claim and/or diagnoses of any type on hospital or outpatient health care claims during the year preceding the index date. We applied a restriction to outpatient claims, such that a comorbid condition could be flagged during the preceding period only if it appeared two or more times at least 30 days apart. We sought to determine the contribution of high glycaemic levels to the risk of severe COVID-19. A high glycaemic level was identified based on the health care service code, which indicated a level of HbA1c greater than or equal to 7% on the last blood sampling^34^.

#### Follow-up and timing

All individuals in the cohort were followed longitudinally through a linked database, which allowed for continuous tracking of their vital status and study outcomes over time. Breakthrough infections were captured until 9 February 2022. Data about hospitalisation were captured until 11 March 2022. For an individual, the follow-up period started on the day after the date of the last vaccine dose received. We measured the time-to-event from the first date after the last vaccination until the primary outcome, death or the end of the study, whichever occurred first.

For the secondary outcome (severe BTI), subjects were followed until the COVID-19 hospitalization date, death, or end of the study, whichever occurred first.

The outcome accrual period started 14 days after the completion of the primary vaccination series (the date of the last vaccine dose) and 14 days after the booster shot for the individuals in the primary + booster dose group.

### Statistical analysis

Incidence rates (IRs) of breakthrough infections were calculated per 10,000 person-days with 95% Poisson confidence intervals. We assessed the distribution of sociodemographic and prevaccination health status to determine differences between fully vaccinated individuals with and without breakthrough infections.

Sociodemographic and comorbidity status data are reported as the means and standard deviations (SDs) for continuous variables and as frequencies and proportions for binary variables.

Education level was missing for 5.5% of participants. We used KNN (k-nearest neighbour) imputation and age, sex, and education level as predictors.

To account for the potential effect of SARS-CoV-2 testing frequency, we estimated the testing propensity for each individual based on negative binomial regression model prediction, using sex, education level, and the natural spline (with four degrees of freedom) of age as covariates.

Factors associated with the outcome (breakthrough infection and related COVID-19 hospitalization) were explored using time-dependent Cox regression, with all those without BTI as a reference group for BTI as an outcome and people who did not require hospitalization as a reference group for severe COVID-19 as an outcome.

We used calendar time as a timescale and binned follow-up period by month to account for differences in overall background infection rates and SARS-CoV-2 strains. Adjusted models included boosting status and time from the last vaccination as time-varying covariates and all other covariates (age, sex, level of education, hospitalization duration during the last year prior to vaccination, Charlson Comorbidity Index, twelve selected diseases, and SARS-CoV-2 testing intensity) as non time-dependent covariates. The results are presented as hazard ratios (HRs) and adjusted hazard ratios (aHRs) with 95% confidence intervals (CIs).

We tested the effect of time since vaccination on BTI by dividing the follow-up period by month and comparing hazards of BTI or hospitalization with COVID-19 in every given period of time and used binned time from the last vaccination as a covariate.

All analyses were performed in R version 4.0.3. We used *tidyverse* package of R statistical software for data pre-processing, the *Epi package* for creating a Lexis object of follow-up, *survival* for survival modeling, and *VIM* for kNN imputation.

## Results

### Study cohort

Among 184,132 individuals in the cohort, 54% (n = 99,453) were females, the mean age was 48.9 years (SD 19.9), and the majority (80.8%) had a high or medium education level. Overall, 84.7% (n = 155,994) of individuals had a CCI score of 0. In general, cardiovascular diseases accounted for one-third (34%) of all comorbid disorders in the present cohort. The most common comorbid condition among the study participants was hypertension (27.8%), followed by mood disorders (7.9%), diabetes (6.6%), heart diseases (6.2%), and chronic lung diseases (4.5%).

Over the period of the study follow-up, 392,647 SARS-CoV-2 tests were performed (mean 2.1 tests per person, SD 2.7).

Slightly less than half of the study participants (46.7%) received a booster dose of the SARS-CoV-2 vaccine on average 199 days (SD 33) after completing the primary vaccination course.

Cohort characteristics are presented in Table 1.

**Table 1:** Characteristics of the study cohort and individuals with SARS-CoV-2 breakthrough infections and severe breakthrough infections in Estonia for the period of 19 January 2021 to 9 February 2022.

	All vaccinated individuals n = 184,132	Breakthrough infections n = 29,688		Severe breakthrough infections n = 355	
	n (%)	n (%)	IR (95% CI)	n (%)	IR (95% CI)
Sociodemographic characteristics					
Female sex (n, %)	99,453 (54.0)	16,532 (55.7)	8.0 (7.88⎼8.12)	176 (49.6)	0.083 (0.071⎼0.096)
Male sex (n, %)	84,679 (46.0)	13,156 (44.3)	8.09 (7.95⎼8.23)	179 (50.4)	0.107 (0.092⎼0.124)
Age, years, mean (SD)	48.9 (19.9)	41.2 (17.3)		68.3 (17.8)	
Age groups (years)					
12⎼39	65,067 (35.3)	14,509 (48.9)	13.21 (13.0⎼13.43)	30 (8.5)	0.026 (0.018⎼0.038)
40⎼49	29,702 (16.1)	6445 (21.7)	11.24 (10.97⎼11.52)	23 (6.5)	0.039 (0.025⎼0.058)
50⎼59	28,512 (15.5)	4121 (13.9)	6.86 (6.66⎼7.08)	37 (10.4)	0.060 (0.042⎼0.083)
60⎼69	27,933 (15.2)	2674 (9.0)	4.32 (4.16⎼4.49)	65 (1.3)	0.103 (0.08⎼0.132)
70⎼79	20,634 (11.2)	1222 (4.1)	2.48 (2.35⎼2.63)	86 (24.2)	0.172 (0.138⎼0.212)
≥ 80	12,284 (6.7)	717 (2.4)	2.31 (2.15⎼2.49)	114 (32.1)	0.354 (0.292⎼0.426)
Education level, n (%)					
Low education level	32,210 (19.1)	5787 (19.5)	9.33 (9.09⎼9.57)	92 (25.9)	0.143 (0.115⎼0.175)
Medium education level	63,412 (35.6)	10,131 (34.1)	7.91 (7.76⎼8.07)	119 (33.5)	0.09 (0.075⎼0.108)
High education level	78,448 (45.2)	13,770 (46.4)	7.68 (7.56⎼7.81)	144 (40.6)	0.078 (0.066⎼0.092)
Health characteristics					
Charlson Comorbidity Index score, n (%)					
CCI score, mean (SD)	0.28 (0.81)	0.1 (0.66)		1.54 (1.85)	
CCI score 0	155,994 (84.7)	26,661 (89.8)	8.75 (8.65⎼8.86)	140 (39.4)	0.045 (0.038⎼0.053)
CCI score 1⎼2	22,886 (12.4)	2520 (8.5)	4.78 (4.59⎼4.97)	135 (38.0)	0.249 (0.209⎼0.295)
CCI score ≥ 3	5252 (2.9)	507 (1.7)	4.22 (3.86⎼ 4.6)	80 (22.5)	0.634 (0.503⎼0.79)
Duration of hospitalization in 365 days pre-vaccination (days), mean (SD)	0.680 (4.5)	0.5 (3.5)		4.75 (13.48)	
Comorbid conditions n (%)					
Diabetes	12,070 (6.6)	1159 (3.9)	4.14 (3.91⎼4.39)	99 (27.9)	0.345 (0.281⎼0.42)
Poorly controlled diabetes (HbA1c ≥ 7)	2511 (1.4)	223 (0.75)	3.88 (3.38⎼4.42)	21 (5.9)	0.357 (0.221⎼0.546)
Obesity	4818 (2.6)	638 (2.1)	6.1 (5.63⎼6.59)	25 (7.0)	0.233 (0.151⎼0.344)
Mood disorders	14,594 (7.9)	2325 (7.8)	7.55 (7.25⎼7.87)	31 (8.7)	0.098 (0.067⎼0.139)
Dementia	1059 (0.58)	116 (0.39)	4.75 (3.92⎼5.69)	15 (4.2)	0.544 (0.305⎼0.898)
Hypertension	51,111 (27.8)	5101 (17.2)	4.33 (4.22⎼4.45)	235 (66.2)	0.195 (0.171⎼0.222)
Heart diseases	11,500 (6.2)	1010 (3.4)	3.72 (3.49⎼3.96)	112 (31.5)	0.399 (0.328⎼0.480)
Cerebrovascular diseases	1671 (0.91)	162 (0.55)	4.14 (3.52⎼4.82)	23 (6.5)	0.566 (0.359⎼0.849)
Cancers	7680 (4.2)	721 (2.4)	3.96 (3.67⎼4.26)	61 (17.2)	0.326 (0.249⎼0.418)
Chronic lung diseases	8323 (4.5)	1167 (3.9)	6.33 (5.97⎼6.71)	61 (17.2)	0.322 (0.246⎼0.413)
Renal diseases	2114 (1.1)	222 (0.75)	4.52 (3.95⎼5.16)	41 (11.5)	0.796 (0.571⎼1.08)
Liver diseases	1365 (0.74)	190 (0.64)	6.7 (5.78–7.72)	12 (3.4)	0.409 (0.211⎼0.714)
Rheumatic diseases	2981 (1.6)	400 (1.3)	5.81 (5.25⎼6.41)	17 (4.8)	0.241 (0.141⎼0.386)
COVID-19 vaccination					
Booster dose	86,056 (46.7)	5129 (17.3)	2.37 (2.3⎼2.43)	28 (7.9)	0.013 (0.009⎼0.019)
No booster	98,076 (53.3)	24,559 (82.7)	16.08 (15.88⎼16.28)	327 (92.1)	0.202 (0.181⎼0.225)

### SARS-CoV-2 breakthrough infections and COVID-19 hospitalizations

Over the follow-up of 11 months, 29,688 individuals had BTIs (IR 8.03, 95% CI 7.95⎼8.13 per 10,000 person-days), and 355 needed hospitalization due to COVID-19 (IR 0.093, 95% CI 0.084⎼0.104 per 10,000 person-days). The median follow-up time to BTI was 212 days (interquartile range, IQR 100, range 0⎼386 days) and 243 days to COVID-19 hospitalization (IQR 100, range 3⎼416 days).

Among individuals without a booster dose (n = 98,076), 24,559 (25%) had BTIs, and 327 (0.33%) were hospitalized for COVID-19. Of those who received a booster vaccine dose (n = 86,056), 6.0% (n = 5,129) had BTIs, and 0.03% (n = 28) were hospitalized for COVID-19.

Of the 355 individuals hospitalized for COVID-19, 15.2% (n = 54) died during hospitalization (crude mortality rate 15.2 per 10,000 hospitalized patients). Those individuals who died during hospitalization were older (77,3 vs 66,3, p < 0.001), and a higher proportion of them had two or more chronic diseases (46.3% vs 18.3%, p < 0.001), cancer (31.5% vs 14.6%, p < 0.01), or renal disease (20.4% vs 10%, p < 0.05).

Detailed data on the effect of different vaccine types and vaccine products on the incidence rates of the main outcomes are presented in the supplementary materials (see Appendix C).

### Factors associated with SARS-CoV-2 breakthrough infection

BTI risk was dependent on having had a booster dose and the time since vaccination.

The risk for BTI began to increase from 2 months after the last vaccination (aHR 1.21, 95% CI 1.16⎼1.27), increasing gradually up to 4 months post-vaccination (aHR 1.75, 95% CI 1.66⎼1.83). Estimates of associations with BTI and COVID-19 hospitalization are presented in Table 2.

**Table 2:** Multivariable analysis of patient characteristics associated with SARS-CoV-2 breakthrough infection and severe breakthrough infection in Estonia for the period of 19 January 2021 to 9 February 2022.

	Breakthrough infection		Severe COVID-19	
	HR (95% CI)	aHR (95% CI)^1^	HR (95% CI)	aHR (95% CI)^1^
Sociodemographic characteristics				
Sex (females, %)	1.07 (1.04–1.09)	1.12 (1.09–1.15)	0.80 (0.65⎼0.99)	0.68 (0.55⎼0.84)
Age groups				
< 40	Ref.^2^		Ref.	
40–49	0.96 (0.93⎼0.98)	1.02 (0.98⎼1.07)	1.61 (0.93⎼2.77)	1.48 (0.86⎼2.57)
50–59	0.61 (0.59–0.63)	0.75 (0.69⎼0.82)	2.63 (1.63⎼4.27)	2.06 (1.25⎼3.42)
60–69	0.39 (0.38–0.41)	0.55 (0.48⎼0.62)	4.69 (3.04⎼7.24)	2.85 (1.76⎼4.61)
70–79	0.24 (0.22⎼0.25)	0.34 (0.29⎼0.39)	8.23 (5.42⎼12.49)	3.87 (2.37⎼6.32)
≥ 80	0.24 (0.22⎼0.26)	0.31 (0.27⎼0.36)	18.04 (12.03⎼27.04)	7.23 (4.36⎼12.02)
Education level				
Low education level	Ref.		Ref.	
Medium education level	0.91 (0.88–0.94)	0.89 (0.86–0.92)	0.66 (0.5–0.87)	0.91 (0.69⎼1.21)
High education level	0.97 (0.94–0.99)	0.9 (0.85⎼0.95)	0.60 (0.46⎼0.78)	0.83 (0.63⎼1.09)
Health characteristics				
Charlson Comorbidity Index score				
CCI score 0	*ref*		*ref*	
CCI score 1–2	0.625 (0.6–0.65)	1.04 (0.98–1.1)	6.25 (4.93⎼7.92)	2.09 (1.54⎼2.83)
CCI score ≥ 3	0.57 (0.52–0.62)	1.18 (1.05⎼1.33)	16.25 (12.34⎼21.4)	2.23 (1.43⎼3.5)
Hospitalization history				
Hospitalisation during the last year (weeks)	0.91 (0.89–0.94)	0.98 (0.96–1.01)	1.13 (1.11⎼1.16)	1.11 (1.07⎼1.15)
Comorbidities				
Diabetes	0.56 (0.53–0.59)	0.98 (0.91⎼1.05)	5.23 (4.15⎼6.6)	1.71 (1.29⎼2.27)
Poorly controlled diabetes (HbA1c ≥ 7)	0.52 (0.46–0.6)	0.85 (0.74⎼0.98)	4.33 (2.79⎼6.74)	1.14 (0.71⎼1.85)
Obesity	0.80 (0.74–0.87)	0.97 (0.89⎼1.05)	2.77 (1.84⎼4.16)	1.05 (0.68⎼1.61)
Mood disorders	0.98 (0.94⎼1.03)	0.98 (0.94⎼1.03)	1.08 (0.75⎼1.56)	0.92 (0.63⎼1.33)
Dementia	0.78 (0.65⎼0.93)	1.69 (1.40⎼2.04)	6.89 (4.09⎼11.61)	1.52 (0.88⎼2.61)
Hypertension	0.51 (0.5⎼0.53)	1.03 (0.99⎼1.07)	4.77 (3.83⎼5.95)	1.11 (0.83⎼1.49)
Heart diseases	0.52 (0.49–0.55)	1.10 (1.02⎼1.18)	6.51 (5.2⎼8.15)	1.34 (1.02⎼1.76)
Cerebrovascular diseases	0.6 (0.51–0.69)	1.15 (0.98⎼1.35)	7.1 (4.65⎼10.85)	1.72 (1.11⎼2.67)
Cancers	0.55 (0.51–0.6)	0.98 (0.90–1.07)	4.46 (3.38⎼5.88)	1.42 (1.04⎼1.94)
Chronic lung diseases	0.86 (0.81⎼0.91)	1.14 (1.07⎼1.22)	4.22 (3.2⎼5.56)	1.72 (1.28⎼2.31)
Renal diseases	0.66 (0.58–0.75)	1.16 (1.01⎼1.34)	10.66 (7.69⎼14.78)	1.92 (1.32⎼2.8)
Chronic liver diseases	0.87 (0.75⎼0.99)	1.06 (0.92⎼1.23)	4.69 (2.64⎼8.35)	2.0 (1.11⎼3.61)
Rheumatic diseases	0.81 (0.73⎼0.89)	1.06 (0.95⎼1.17)	2.89 (1.78⎼4.71)	1.57 (0.95⎼2.59)
Predicted testing propensity	1.88 (1.85⎼1.91)	1.25 (1.15⎼1.34)		
COVID-19 vaccination				
Booster dose	0.41 (0.4–0.42)	0.95 (0.9–0.99)	0.54 (0.35–0.84)	0.32 (0.19–0.54)

^1^Adjusted for sex, age groups, boosting, *Charlson Comorbidity Index*, hospital stay duration before vaccination, level of education, comorbidities, time since last vaccination group and intensity of testing.

^2^*Ref* reference group.

**Figure 1:**
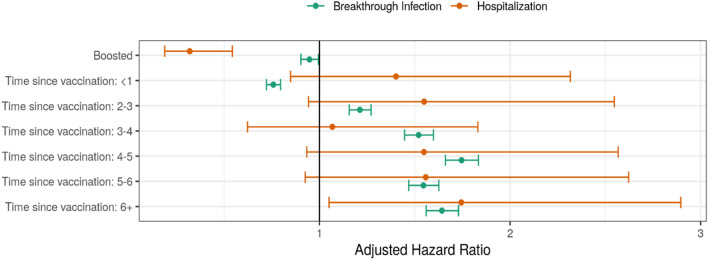
Time since vaccination and effect of boosting on the risk of BTI and COVID-19-associated hospitalization (aHR with 95% CI).

Having had a booster dose of vaccine demonstrated a weak protective effect against BTI (aHR 0.95, 95% CI 0.90⎼0.99) (Fig. 1).

In addition, BTI risk was associated with sex, age, education level, number of comorbidities, and specific comorbidities. Females were more susceptible to BTI than males (aHR 1.12, 95% CI 1.09⎼1.15). Those aged 50 and older had lower risks for BTI than those younger than 40 years (aHR 0.75, 95% CI 0.69⎼ 0.82). We observed an inverse dose–response effect of age on the risk of BTI (decreased risk with increasing age); i.e., among people older than 70 years, there was an aHR of 0.34 (95% CI 0.29⎼0.39) for the 70–79 age group and an aHR of 0.31 (95% CI 0.27⎼0.36) for those aged 80 or older.

Only those with three or more comorbid conditions (CCI score ≥ 3) had a greater risk for breakthrough infection (aHR 1.18, 95% CI 1.05⎼1.33).

People with poor glycaemic control had a slightly lower risk of BTI (aHR 0.85, 95% CI 0.74⎼0.98). Individuals with renal diseases (aHR 1.16, 95% CI 1.01⎼1.34), heart diseases (aHR 1.10, 95% CI 1.02⎼1.18), and dementia (aHR 1.69, 95% CI 1.40⎼2.04) were associated with an increased risk for BTI.

### Factors associated with severe breakthrough infection

The risk for severe BTI began to rise after 6 months post-vaccination, achieving an almost twofold increased risk for severe BTI in the adjusted analysis (aHR 1.74, 95% CI 1.05⎼2.90).

Boosted individuals had a threefold lower risk of hospitalization associated with severe BTI (aHR 0.32, 95% CI 0.19–0–0.54) (see Fig. 1).

Females had a lower risk of severe BTI, which was expressed by substantially lower risks of hospitalization (aHR 0.68, 95% CI 0.55⎼0.84). The risk for severe BTI began to rise from the age of 50 years, increasing gradually with every decade of life and achieving an almost sevenfold increase in risk for those aged 80 and older (aHR 7.23, 95% CI 4.36⎼12.02). A greater CCI score was associated with a greater risk of severe BTI. Thus, vaccinated individuals with CCI scores of 1 or 2 had at least a twofold risk (aHR 2.09, 95% CI 1.54⎼2.83) of developing severe disease during BTI, while a greater CCI score led to an even greater risk for severe BTI (aHR 2.23, 95% CI 1.43⎼3.50 for those people with a CCI score ≥ 3). Among comorbidities, renal diseases demonstrated the most pronounced effect on the risk of hospitalization during BTI (aHR 1.92, 95% CI 1.32⎼2.80); other chronic diseases associated with elevated risk were chronic lung diseases (aHR 1.72, 95% CI 1.28⎼2.31), cerebrovascular diseases (aHR 1.72, 95% CI 1.11⎼2.67), diabetes (aHR 1.71, 95% CI 1.29⎼2.27), heart diseases (aHR 1.34, 95% CI 1.02⎼1.76), and cancers (aHR 1.42, 95% CI 1.04⎼1.94).

## Discussion

We followed a large, population-based cohort of SARS-CoV-2-vaccinated, infection-naive individuals. The study results demonstrated a remarkable and consistent protective effect of vaccination against severe COVID-19 for up to six months, with the booster dose offering an additional pronounced benefit (up to 81% lower hospitalization risk compared to the primary vaccination series alone). The risk of severe BTI requiring hospitalisation began to rise at 50 years of age and increased constantly with each subsequent decade of life. Sex-specific differences and unfavorable impact of chronic diseases, especially those affecting cardiovascular, lung, or renal endothelia, were observed. To our knowledge, this is one of the few retrospective cohort studies aimed at assessing the risk factors for severe COVID-19 among people vaccinated against SARS-CoV-2.

**Figure 2:**
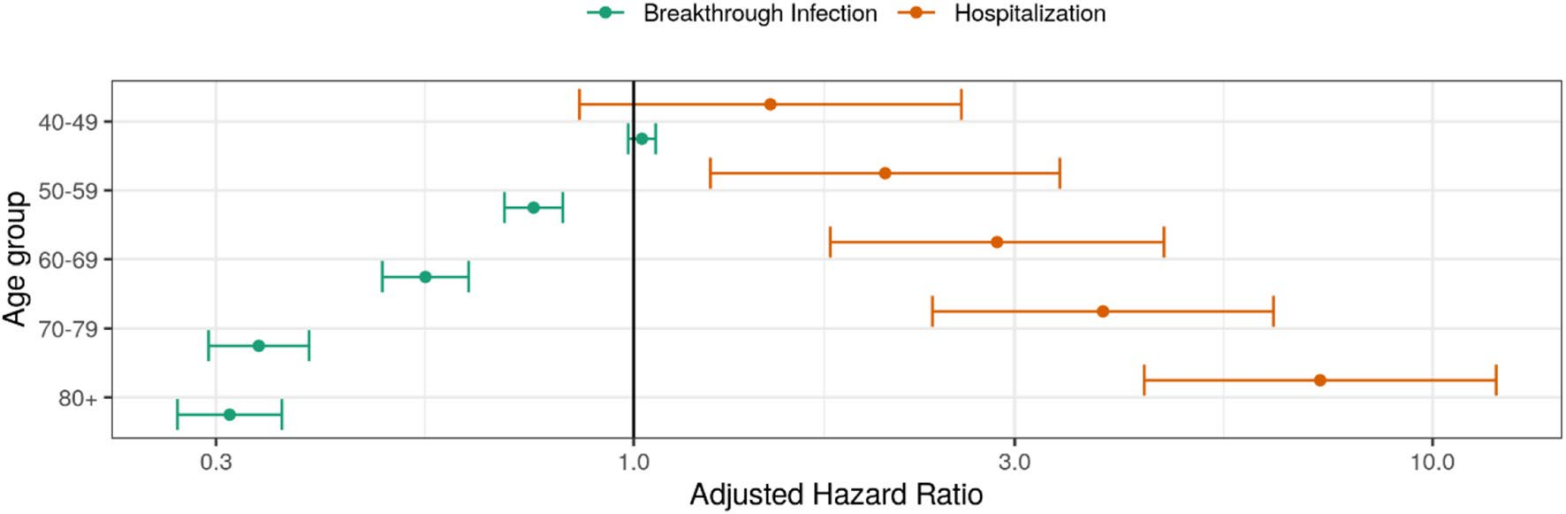
Risk for SARS-CoV-2 breakthrough infection and severe COVID-19 according to age of study participants.

Our analysis revealed that age had a bidirectional effect on BTI: we observed a decreased risk of infection and an increased risk of severe disease with increasing age in the case of BTI (see Fig. 2). This was in concordance with other studies that demonstrated a lower risk of BTI with increasing age^35^. Porru et al. found a similar effect of age on the risk of BTI in a large European multicentre study^36^. This finding may be related to the reduced number of social contacts and stricter adherence to pandemic-related restrictions among older people, as well as expansion of testing strategy to younger age groups during the second and third waves of pandemic. The effect of age on the risk of BTI in our study was the opposite of that demonstrated in risk assessment studies conducted in the pre-vaccination era. Additionally, there is some variability in terms of the incidence of BTI in studies conducted during the same period of the pandemic, depending on the prevalent SARS-CoV-2 strain and setting^37,38^.

Our analysis revealed that while the protective effect of vaccination against breakthrough infection waned over time, the occurrence of severe disease was delayed for at least six months. This finding of a strong protective effect of vaccination against hospitalization is in concordance with other studies, revealing an immense benefit of booster doses on overall vaccine effectiveness during the first two years of the pandemic^39–41^. Compared to the historical cohort^42^, the COVID-19 hospitalization rate among vaccinated people with BTIs was at least five times lower compared to the hospitalization rate in the prevaccination era and during the first two waves of the pandemic. The mortality rate among vaccinated individuals requiring COVID-19 hospitalization in our study was lower than that among unvaccinated people in previous studies^43^. That, in conjunction with decreased hospitalization risk, makes the effect obtained from the booster dose undeniably important.

We identified important individual characteristics associated with the risk of severe COVID-19 among vaccinated individuals. In our study males were significantly more susceptible to severe BTI, similar to previous studies that included unvaccinated people^44^.

Studies released in the pre-vaccination era reported a close relationship between different range of cardiovascular diseases and severe COVID-19, including the pronounced influence of isolated hypertension on the risk of hospitalisation and death in a study engaged historical cohort of unvaccinated individuals in Estonia^42^. Whether this also applies to vaccinated individuals warrants careful exploration. In our study, we did not find an association between hypertension and the risk of COVID-19-related hospitalization. However, those with ischaemic or structural heart disease were at risk for COVID-19 hospitalization. This connection is quite predictable, considering the devastating effect of respiratory viruses on the endothelium of small intramyocardial and coronary heart vessels. This process, induced by the response of the innate immune system to viral replication in the host body, plays an important role in the pathogenesis of infectious heart damage, formation of microthrombosis, and coronary vasospasm, which may lead to deterioration of all vital functions in infected organisms^45^.

Contrary to previous studies that demonstrated the close relationship between dementia and the risk of poor SARS-CoV-2 breakthrough infection outcome, we did not find the same association^46^. In our study, dementia was a risk factor for breakthrough infection itself but not for severe COVID-19.

Marfella et al. reported that HbA1c ≥ 7 in the postvaccination period is associated with lower immune responses and an increased risk of SARS-CoV-2 breakthrough infections in diabetic patients^47^. Our study failed to show an association between poor glycaemic control in the prevaccination period and the risk of BTI. Risk factors for severe outcome among vaccinated individuals in our study generally resembled those reported in risk assessment studies in the prevaccination era^48,49^.

We assume that population size, racial differences, discrepancies in testing policy, and hospitalization have all significant impact on the results of different studies. These factors need to be carefully considered when interpreting results across studies.

Based on the data of our study, we can recommend that an additional shot of SARS-CoV-2 vaccine should be given to those at greater risk of a complicated COVID-19 course no later than 6 months after the initial vaccination course. There are people with identifiable characteristics at higher risk for severe BTIs. This can help health authorities establish and manage vaccination schedules.

### Limitations and strengths

Our analysis used data derived from the nationwide and population-based universal tax-funded health care system. We believe our findings are firm given the sample size and the completeness of the data obtained from nationwide electronic health data. The findings of our research have good external validity due to universal SARS-CoV-2 PCR testing strategies and the high coverage of the study population by testing.

It is likely that the BTI incidence we describe here is an underestimate. Not all (asymptomatic, mild disease) COVID-19 cases undergo testing, which may lead to an underestimation of the true incidence among those with lower testing propensity. However, we are more confident in the estimate of severe disease from this study, as this is not open to SARS-CoV-2 testing-related misclassification.

Our inclusion of four of the most widely used vaccines for primary and booster vaccinations together increases the generalizability of our findings.

Our study was missing information on cohort members’ lifestyles (i.e., smoking, alcohol consumption, daily physical activity) and medication use. Therefore, our estimates can be affected by residual confounding. However, we attempted to minimize this by adjusting for the most well-documented risk factors for SARS-CoV-2 infection and severe COVID-19 (health, vaccination, sociodemographic characteristics). To alleviate bias related to differences in testing behavior among those with and without breakthrough infections, we used estimated individual testing intensity as a covariate in the model.

During the study period, the alpha and delta strains of SARS-CoV-2 were prevalent in Estonia^22^. We could not accurately assess the impact of different SARS-CoV-2 variants on the main outcomes. To gauge bias related to differences in the virulence and pathogenicity of different SARS-CoV-2 strains, we accounted for prevalent strains and background infection rates and then binned the observation period for each individual on the basis of calendar month. By doing this, we ensured that people at risk at any given period were similar with respect to the strain and infection rate.

Whether our results are also robust in the case of the evolving SARS-CoV-2 strains is currently unclear. Additionally, assessing the need and timing of repeated continuous boosting is an urgent research need.

Vaccination remains an effective tool for preventing severe COVID-19 and its complications, even in the face of emerging variants and breakthrough infections. Combating COVID-19 is an iterative and ongoing process, so monitoring vaccination-related protection is essential to managing the epidemic.

Health authorities should continue to encourage COVID-19 vaccination for everyone who is eligible. Considering the high ability of SARS-CoV-2 to mutate and its associated ability to evade the immune response, it is necessary to adjust government-implemented vaccination strategies based on the latest knowledge about the risk of breakthrough infections. Additionally, a more individualized approach should be taken during the planning of preventive strategies. Additional protective measures and elaborated treatment strategies might be necessary for those at risk of severe breakthrough infection.

Research should focus on the exploration of additional risk factors that contribute to severe COVID-19 outcome, such as different kinds of early interventions, and how their implementation alters the risk of severe SARS-CoV-2 breakthrough infection, to plan targeted strategies for those who are most vulnerable.

## Conclusion

The protective effect of vaccination again severe COVID-19 remains considerable for up to six months. However, there are still vulnerable groups among vaccinated people who need additional protection due to the ongoing threat of SARS-CoV-2 infection. Veracious data on health inequalities in subgroups are essential for developing local and nationwide COVID-19 preventive measures and treatment guidelines.

## Supplementary Information

Supplementary Information 1.

Supplementary Information 2.

Supplementary Information 3.

## Data Availability

The data that support the findings of this study are available from the EHB, EHIB, and the Causes of Death Registry but restrictions apply to the availability of these data, which were used under license for the current study, and so are not publicly available. Data are however available from the corresponding author (tatjana.meister@ut.ee) upon reasonable request and with permission of EHB, EHIB, and the Causes of Death Registry.

## References

[1] COVID-19 vaccination tracker. https://graphics.reuters.com/world-coronavirus-tracker-and-maps/vaccinationrollout-and-access/ [Accessed by 25 June 2022].

[2] European Centre for Disease Prevention and Control. *Interim analysis of COVID-19 vaccine effectiveness against Severe Acute Respiratory Infection due to laboratory-confirmed SARS-CoV-2 among individuals aged 65 years and older, ECDC multi-country study*. ECDC: Stockholm (2021)

[3] Overview of COVID-19 vaccination strategies and vaccine deployment plans in the EU/EEA and the UK. December 2020. https://www.ecdc.europa.eu/sites/default/files/documents/Overview-of-EU_EEA-UK-vaccination-deployment-plans.pdf [Assessed by 5 October 2022].

[4] WHO SAGE roadmap for prioritizing uses of COVID-19 vaccines in the context of limited supply: an approach to inform planning and subsequent recommendations based on epidemiological setting and vaccine supply scenarios, first issued 20 October 2020, latest update 16 July 2021. [Assessed by 5 October 2022].

[5] Deb P (2022). The effects of COVID-19 vaccines on economic activity. Swiss J. Econ. Stat..

[6] WHO Coronavirus (COVID-19) Dashboard. https://covid19.who.int/ [Accessed by 08 September 2022].

[7] Fenemigho I (2020). COVID-19, flattening the curve: Recommendations towards control and managing a second wave. J. Glob. Health Rep..

[8] Statement on the tenth meeting of the International Health Regulations (2005) Emergency Committee regarding the coronavirus disease (COVID-19) pandemic https://www.who.int/news/item/19-01-2022-statement-on-the-tenth-meeting-of-the-international-health-regulations-(2005)-emergency-committee-regarding-the-coronavirus-disease-(covid-19)-pandemic [Assessed by 15 October 2022].

[9] Higdon M (2022). Duration of effectiveness of vaccination against COVID-19 caused by the omicron variant. Lancet Infect. Dis..

[10] Edara V (2022). mRNA-1273 and BNT162b2 mRNA vaccines have reduced neutralizing activity against the SARS-CoV-2 omicron variant. Cell. Rep. Med..

[11] Pulliam, J.R.C. *et al*. Increased risk of SARS‐CoV‐2 reinfection associated with emergence of the Omicron variant in South Africa. *medRxiv*. 10.1101/2021.11.11.21266068 (2021).

[12] Wang R, Chen J, Gao K, Wei GW (2021). Vaccine-escape and fast-growing mutations in the United Kingdom, the United States, Singapore, Spain, India, and other COVID-19-devastated countries. Genomics.

[13] Laboratory-Confirmed COVID-19–Associated Hospitalizations Among Adults During SARS-CoV-2 Omicron BA.2 Variant Predominance—COVID-19–Associated Hospitalization Surveillance Network, 14 States, June 20, 2021–May 31, 2022. Centers for Disease Control and Prevention. https://www.cdc.gov/mmwr/volumes/71/wr/mm7134a3.htm. [Accessed by September 08, 2022]. 10.15585/mmwr.mm7134a3 PMC9422959 36006841

[14] Lee CJ (2022). Clinical manifestations of COVID-19 breakthrough infections: A systematic review and meta-analysis. J. Med. Virol..

[15] COVID-19 Cases, Hospitalizations, and Deaths by Vaccination Status Washington State Department of Health October 12, 202. https://doh.wa.gov/sites/default/files/2022-02/421-010-CasesInNotFullyVaccinated.pdf [Assessed by 15 October 2022].

[16] Havers FP (2022). COVID-19-associated hospitalizations among vaccinated and unvaccinated adults 18 years or older in 13 us states, January 2021 to April 2022. JAMA Intern. Med..

[17] Hansen CH, Michlmayr D, Gubbels SM, Mølbak K, Ethelberg S (2021). Assessment of protection against reinfection with SARS-CoV-2 among 4 million PCR-tested individuals in Denmark in 2020: A population-level observational study. Lancet.

[18] Gazit, S. *et al.* Comparing SARS-CoV-2 natural immunity to vaccine-induced immunity: reinfections versus breakthrough infections. medRxiv 2021.08.24.21262415.

[19] Kuhlmann, C. *et al.* Breakthrough infections with SARS-CoV-2 omicron despite mRNA vaccine booster dose [published correction appears in Lancet. 2022 Feb 12;399(10325):628]. *Lancet*. **399**(10325), 625–626 (2022). 10.1016/S0140-6736(22)00090-3 PMC8765759 35063123

[20] Loconsole D (2022). Autochthonous outbreak of SARS-CoV-2 omicron variant in booster-vaccinated (3 Doses) Healthcare Workers in Southern Italy: Just the Tip of the Iceberg?. Vaccines.

[21] https://www.strobe-statement.org/ [accessed by 13 May 2023].

[22] COVID-19 Data Portal, Estonia. https://covid19dataportal.ee/genomics_transcriptomics/ [accessed by 08 September 2022].

[23] Official information on COVID-19 vaccination in Estonia. https://vaktsineeri.ee/en/covid-19/statistics/ [Assessed by 2 August 2022].

[24] The Health and Welfare Information Systems Centre (TEHIK). https://www.tehik.ee/en/about [Accessed by 24 May 2022].

[25] e-Health Record https://e-estonia.com/solutions/healthcare/e-health-records/ [Accessed by 01 February 2022].

[26] Estonian Health Insurance Fund. https://www.haigekassa.ee/en/people/health-insurance [Accessed by 08 September 2022].

[27] Kluberg SA (2022). Validation of diagnosis codes to identify hospitalized COVID-19 patients in health care claims data. Pharmacoepidemiol. Drug Saf..

[28] ICD-10-CM official coding and reporting guidelines April 1, 2020 through September 30, 2020. Centers for Disease Control and Prevention National Center for Health Statistics. https://www.cdc.gov/nchs/data/icd/COVID-19-guidelines-final.pdf [Accessed by 09 September 2022].

[29] http://uis.unesco.org/en/topic/international-standard-classification-education-isced [Assessed by 28 July 2022].

[30] International Standard Classification of Education (ISCED). https://ec.europa.eu/eurostat/statistics-explained/index.php?title=International_Standard_Classification_of_Education_(ISCED) [Accessed by 09 September 2022].

[31] Comoglu S, Kant A (2022). Does the Charlson comorbidity index help predict the risk of death in COVID-19 patients?. North Clin. Istanb..

[32] Quan H (2011). Updating and validating the Charlson comorbidity index and score for risk adjustment in hospital discharge abstracts using data from 6 countries. Am. J. Epidemiol..

[33] Lingsma HF (2018). Evaluation of hospital outcomes: the relation between length-of-stay, readmission, and mortality in a large international administrative database. BMC Health Serv. Res..

[34] Family physicians’ quality system indicator descriptions 2017. Estonian Health Insurance Fund. https://haigekassa.ee/en/partner/primary-health-care-quality-system [Accessed by 10 October 2022].

[35] Chadeau-Hyam M (2022). SARS-CoV-2 infection and vaccine effectiveness in England (REACT-1): A series of cross-sectional random community surveys. Lancet Respir. Med..

[36] Porru S (2022). SARS-CoV-2 breakthrough infections: Incidence and risk factors in a large European multicentric cohort of health workers. Vaccines (Basel)..

[37] Stouten V (2022). Incidence and risk factors of COVID-19 vaccine breakthrough infections: A prospective cohort study in Belgium. Viruses.

[38] Almufty HB, Mamani MMA, Ali AH, Merza MA (2022). COVID-19 vaccine breakthrough infection among fully vaccinated healthcare workers in Duhok governorate, Iraqi Kurdistan: A retrospective cohort study. J. Med. Virol..

[39] Oster Y, Benenson S, Nir-Paz R, Buda I, Cohen MJ (2022). The effect of a third BNT162b2 vaccine on breakthrough infections in health care workers: A cohort analysis. Clin. Microbiol. Infect..

[40] Effectiveness of 2, 3, and 4 COVID-19 mRNA Vaccine Doses Among Immunocompetent Adults During Periods when SARS-CoV-2 Omicron BA.1 and BA.2/BA.2.12.1 Sublineages Predominated — VISION Network, 10 States, December 2021–June 2022. Centers for Disease control and Prevention. https://www.cdc.gov/mmwr/volumes/71/wr/mm7129e1.htm?s_cid=mm7129e1_w [Assessed by 8 September 2022].

[41] Tartof SY (2022). Effectiveness of a third dose of BNT162b2 mRNA COVID-19 vaccine in a large US health system: A retrospective cohort study. Lancet Reg. Health Am..

[42] Meister T (2022). Clinical characteristics and risk factors for COVID-19 infection and disease severity: A nationwide observational study in Estonia. PLoS ONE.

[43] Macedo, A., Gonçalves, N., & Febra, C. COVID-19 fatality rates in hospitalized patients: systematic review and meta-analysis. Ann. Epidemiol. **57**, 14–21 (2021), ISSN 1047-2797. 10.1016/j.annepidem.2021.02.012 PMC7920817 33662494

[44] Peckham H, de Gruijter NM, Raine C (2020). Male sex identified by global COVID-19 meta-analysis as a risk factor for death and ITU admission. Nat. Commun..

[45] Blauwet, L.A, & Cooper, L.T. Myocarditis. Prog. Cardiovasc. Dis. **52**(4), 274–88 (2010). 10.1016/j.pcad.2009.11.006 PMC5951175 20109598

[46] Wang, L., Davis, P.B., Kaelber, D.C., Xu, R. COVID-19 breakthrough infections and hospitalizations among vaccinated patients with dementia in the United States between December 2020 and August 2021 [published online ahead of print, 2022 Apr 13]. *Alzheimers Dement*. 10.1002/alz.12669 (2022). 10.1002/alz.12669 PMC9073984 35417628

[47] Marfella R (2022). Glycaemic control is associated with SARS-CoV-2 breakthrough infections in vaccinated patients with type 2 diabetes. Nat. Commun..

[48] Villar-García, J. *et al.* Risk factors for SARS-CoV-2 infection, hospitalisation, and death in Catalonia, Spain: a population-based cross-sectional study. medRxiv 2020.08.26.20182303.

[49] Pijls BG (2021). Demographic risk factors for COVID-19 infection, severity, ICU admission and death: A meta-analysis of 59 studies. BMJ Open.

