# Supplementary material for: A retrospective cohort study of incidence and risk factors for severe SARS-CoV-2 breakthrough infection among fully vaccinated people
Source: Sci Rep. 2023 May 26;13:8531. doi: 10.1038/s41598-023-35591-w (PMC10213588; doi:10.1038/s41598-023-35591-w)
Supplement: Supplementary file 1 — Supplementary Information 1. [file 41598_2023_35591_MOESM1_ESM.docx]

Appendix A. Flow-chart of study cohort

Inclusion criteria

- ≥12 years old
- not previously tested positive for SARS-CoV-2
- received at least one dose of SARS-CoV-2 vaccine in the period of 27 Dec 2020 ⎼ 08 Feb 2022

Study vaccinated cohort

(n=184,132)

*Fully vaccinated by 19th of January 2021*

Severe BTI

(n=327)

Severe BTI

(n=28)

Breakthrough infection (BTI)

(n=24,559)

Breakthrough infection (BTI)

(n=5,129)

Individuals received only primary series of SARS-CoV-2 vaccine

(n=98,076)

Individuals received booster of SARS-CoV-2 vaccine

(n=86,056)

Excluded:

- incomplete primary series of SARS-CoV-2 vaccination
- positive SARS-CoV-2 PCR < 14 days after primary vaccine series
- vaccinated <14 days before the end of the study period

(n= 66,370)

Source vaccinated cohort

(n= 250,502)
