# Supplementary material for: A retrospective cohort study of incidence and risk factors for severe SARS-CoV-2 breakthrough infection among fully vaccinated people
Source: Sci Rep. 2023 May 26;13:8531. doi: 10.1038/s41598-023-35591-w (PMC10213588; doi:10.1038/s41598-023-35591-w)
Supplement: Supplementary file 2 — Supplementary Information 2. [file 41598_2023_35591_MOESM2_ESM.docx]

Appendix B

ICD-10 codes used to define underlying conditions and risk groups in multivariable analysis.

| **Co-morbidity** | **ICD-10 codes** |
| --- | --- |
| Diabetes | E10⎼E11[^[[1]](#endnote-1)^] |
| Obesity | E66 [^[[2]](#endnote-2)^,^[[3]](#endnote-3)^,^[[4]](#endnote-4)^] |
| Mood disorders | F30⎼F39 and F40⎼F48 [^[[5]](#endnote-5)^] |
| Dementia | F00⎼F03, G30, G31.0, G31.1, G31.8, G31.9 [^[[6]](#endnote-6)^] |
| Hypertension | I10⎼I15 [^[[7]](#endnote-7)^] |
| Heart diseases | I20-I25, I34 - I37, I42-I43, I50 [^[[8]](#endnote-8)^] |
| Cerebrovascular diseases | G45, G46, I63, I64 [^[[9]](#endnote-9)^] |
| Cancers | C00-C97 [^[[10]](#endnote-10)^] |
| Chronic lung diseases | J41, J42, J43, J44, J45, J47 [^[[11]](#endnote-11)^] |
| Renal diseases | N03, N04, N05, N06, N07, N08, N11, N14-N16, N18, Z99.2, I12 [^[[12]](#endnote-12)^] |
| Liver diseases | K70-77 [^[[13]](#endnote-13)^] |
| Rheumatic diseases | M05-M09, M30-M36 [^[[14]](#endnote-14)^] |

1. McGurnaghan SJ at al. Risks of and risk factors for COVID-19 disease in people with diabetes: a cohort study of the total population of Scotland. Lancet Diabetes Endocrinol. 2021 Feb;9(2):82-93 [↑](#endnote-ref-1)
2. Burn E, Tebé C at al. The natural history of symptomatic COVID-19 during the first wave in Catalonia. Nat Commun. 2021 Feb 3;12(1):777 [↑](#endnote-ref-2)
3. Suleyman G at al. Clinical Characteristics and Morbidity Associated With Coronavirus Disease 2019 in a Series of Patients in Metropolitan Detroit. JAMA Netw Open. 2020 Jun 1;3(6):e2012270. [↑](#endnote-ref-3)
4. Booth A at al. Population risk factors for severe disease and mortality in COVID-19: A global systematic review and meta-analysis. PLoS One. 2021 Mar 4;16(3):e0247461. [↑](#endnote-ref-4)
5. Wang Q, Xu R, Volkow ND. Increased risk of COVID-19 infection and mortality in people with mental disorders: analysis from electronic health records in the United States. World Psychiatry. 2021 Feb;20(1):124-130 [↑](#endnote-ref-5)
6. Wang Q, Davis PB, Gurney ME, Xu R. COVID-19 and dementia: Analyses of risk, disparity, and outcomes from electronic health records in the US. Alzheimers Dement. 2021 Aug;17(8):1297-1306 [↑](#endnote-ref-6)
7. Li X at al. Risk factors for severity and mortality in adult COVID-19 inpatients in Wuhan. J Allergy Clin Immunol. 2020 Jul;146(1):110-118 [↑](#endnote-ref-7)
8. Judit Villar-García at al. Risk factors for SARS-CoV-2 infection, hospitalisation, and death in Catalonia, Spain: a population-based cross-sectional study. medRxiv 2020.08.26.20182303 [↑](#endnote-ref-8)
9. Cheng S, Zhao Y, Wang F, Chen Y, Kaminga AC, Xu H. Comorbidities' potential impacts on severe and non-severe patients with COVID-19: A systematic review and meta-analysis. Medicine (Baltimore). 2021 Mar 26;100(12):e24971 [↑](#endnote-ref-9)
10. Meng, Y at al. Cancer history is an independent risk factor for mortality in hospitalized COVID-19 patients: a propensity score-matched analysis. *J Hematol Oncol* **13**, 75 (2020) [↑](#endnote-ref-10)
11. Beltramo G at al. Chronic respiratory diseases are predictors of severe outcome in COVID-19 hospitalised patients: a nationwide study. Eur Respir J. 2021 Dec 9;58(6):2004474 [↑](#endnote-ref-11)
12. Petrilli CM at al. Factors associated with hospital admission and critical illness among 5279 people with coronavirus disease 2019 in New York City: prospective cohort study. BMJ. 2020 May 22;369:m1966 [↑](#endnote-ref-12)
13. Nagarajan R, Krishnamoorthy Y, Rajaa S, Hariharan VS. COVID-19 Severity and Mortality Among Chronic Liver Disease Patients: A Systematic Review and Meta-Analysis. Prev Chronic Dis 2022;19:210228 [↑](#endnote-ref-13)
14. Williamson EJ at al. Factors associated with COVID-19-related death using OpenSAFELY. Nature. 2020 Aug;584(7821):430-436 [↑](#endnote-ref-14)
