# Supplementary material for: A retrospective cohort study of incidence and risk factors for severe SARS-CoV-2 breakthrough infection among fully vaccinated people
Source: Sci Rep. 2023 May 26;13:8531. doi: 10.1038/s41598-023-35591-w (PMC10213588; doi:10.1038/s41598-023-35591-w)
Supplement: Supplementary file 3 — Supplementary Information 3. [file 41598_2023_35591_MOESM3_ESM.docx]

Appendix C

Incidence rates (IR) of SARS-CoV-2 breakthrough infections (BTI) and severe COVID-19 in fully vaccinated individuals in Estonia for the period of 19 Jan 2021 to 9 Feb 2022 by vaccine type and brand.

|  | **All vaccinated individuals**  n=184,132 | **BTI**  n =29,688 | | **Severe COVID-19**  n=355 | |
| --- | --- | --- | --- | --- | --- |
|  | n (%) | n (%) | **IR (95%CI)** | n (%) | **IR (95%CI)** |
| **Primary series of COVID-19 vaccine** | | | | | |
| ***All primary series*** | 98,076 (53·3) | 24,559 (82·7) |  | 327 (92·1) |  |
| *mRNA/mRNA** | 78,384 (42·6) | 18,698 (63) | **15.32**  **(15.1⎼15.54)** | 232 (65,4) | **0.18**  **(0.16⎼0.2)** |
| Comirnaty/Comirnaty | 67,988 (36·9) | 16,790 (56·6) | **15.89**  **(15.65⎼16.13)** | 216 (60·8) | **0.19**  **(0.169⎼0.22)** |
| Comirnaty/Spikevax | 45 (0·0) | 4 (0·0) | **9.97**  **(2.72⎼25.53)** | 0 (0,0) |  |
| Spikevax/Comirnaty | 96 (0·1) | 16(0·1) | **18.83**  **(10.76⎼30.58)** | 0 (0·0) |  |
| Spikevax/Spikevax | 10,255 (5·6) | 1888 (6·4) | **11.6**  **(11.08⎼12.13)** | 16 (4·5) | **0.094**  **(0.054⎼0.153)** |
| *VV/VV*** | 19,354 (10·5) | 5,800 (19·5) | **19.15**  **(18.66⎼19.65)** | 94 (26·5) | **0.292**  **(0.236⎼0.357)** |
| Janssen | 13,353 (7·3) | 2,829 (9·5) | **15.33**  **(14.77⎼15.9)** | 35 (9·9) | **0.18**  **(0.126⎼0.251)** |
| Vaxzevria/Janssen | 4 (0·0) | 0 (0·0) |  | 0 (0·0) |  |
| Vaxzevria/Vaxzevria | 5,997 (3·3) | 2,971 (10·0) | **25.11**  **(24.22⎼26.03)** | 59 (16·6) | **0.46**  **(0.35⎼0.594)** |
| *mRNA/VV or VV/mRNA* | 338 (0·2) | 61 (0·2) | **15.99**  **(12.23⎼20.54)** | 1 (0·3) | **0.25**  **(0.006⎼1.395)** |
| Comirnaty/Janssen | 11 (0·0) | 2 (0·0) | **16.82**  **(2.04⎼60.76)** | 0 (0·0) |  |
| Comirnaty/Vaxzevria | 10 (0·0) | 3 (0·0) | **13.64**  **(2.81⎼39.85)** | 0 (0·0) |  |
| Spikevax/Janssen | 1 (0·0) | 0 (0·0) |  | 0 (0·0) |  |
| Spikevax/Vaxzevria | 2 (0·0) | 0 (0·0) |  | 0 (0·0) |  |
| Vaxzevria/Comirnaty | 271 (0·1) | 49 (0·2) | **16.59**  **(12.27⎼21.93)** | 1 (0·3) | **0.323**  **(0.008⎼1.798)** |
| Vaxzevria/Spikevax | 43 (0·0) | 7 (0·0) | **15.05**  **(6.05⎼31.02)** | 0 (0·0) |  |
| **COVID-19 booster vaccine** | | | | | |
| ***All boosters*** | 86,056 (46·7) | 5,129 (17·3) |  | 28 (7·9) |  |
| *mRNA/mRNA + mRNA* | 59,963 (32·6) | 3,015 (10·2) | **1.97**  **(1.9⎼2.04)** | 25 (7·0) | **0.016**  **(0.01⎼0.024)** |
| Comirnaty/Comirnaty  + Comirnaty | 48,488 (26·3) | 2,516 (8·5) | **2.01**  **(1.93⎼2.09)** | 20 (5·6) | **0.016**  **(0.01⎼0.025)** |
| Comirnaty/Comirnaty  +Spikevax | 4,564 (2·5) | 255 (0·9) | **2.44**  **(2.15⎼2.76)** | 0 (0·0) |  |
| Comirnaty/Spikevax  +Spikevax | 3  0·0 | 1 (0·0) | **16.72**  **(0.42⎼93.17)** | 0 (0·0) |  |
| Comirnaty/Spikevax  +Comirnaty | 5 (0·0) | 0 (0·0) |  | 0 (0·0) |  |
| Spikevax/Comirnaty  +Comirnaty | 17 (0·0) | 2 (0·0) | **4.04**  **(0.49⎼14.6)** | 0 (0·0) |  |
| Spikevax/Comirnaty  +Spikevax | 2 (0·0) | 0 (0·0) |  | 0 (0·0) |  |
| Spikevax/Spikevax  +Comirnaty | 3,100 (1·7) | 100 (0·3) | **1.2**  **(0.98⎼1.46)** | 3 (0·8) | **0.036**  **(0.007⎼0.105)** |
| Spikevax/Spikevax  +Spikevax | 3,784 (2·1) | 141 (0·5) | **1.55**  **(1.31⎼1.83)** | 2 (0·6) | **0.022**  **(0.003⎼0.079)** |
| *mRNA/mRNA+VV* | 8 (0·0) | 2 (0·0) | **8.69**  **(1.05⎼31.4)** | 0 (0·0) |  |
| Comirnaty/Comirnaty  +Janssen | 5 (0·0) | 1 (0·0) | **8.22**  **(0.21⎼45.82)** | 0 (0·0) |  |
| Comirnaty/Comirnaty  +Vaxzevria | 3 (0·0) | 1 (0·0) | **9.22**  **(0.23⎼ 51.35)** | 0 (0·0) |  |
| *VV/VV+mRNA* | 25,945 (14·1) | 2,098 (7·1) | **3.32**  **(3.18⎼3.46)** | 3 (0·8) | **0.005**  **(0.001⎼0.014)** |
| Janssen/Comirnaty | 2,531 (1·4) | 156 (0·5) | **2.77**  **(2.35⎼3.24)** | 0 (0·0) |  |
| Janssen/Spikevax | 902 (0·5) | 47 (0·2) | **2.38**  **(1.75⎼3.16)** | 0 (0·0) |  |
| Vaxzevria/Janssen  +Spikevax | 1 (0·0) | 0 (0·0) |  | 0 (0·0) |  |
| Vaxzevria/Vaxzevria  +Comirnaty | 18768 (10·2) | 1621 (5·5) | **3.47**  **(3.31⎼3.65)** | 3 (0·8) | **0.006 (0.001⎼0.019)** |
| Vaxzevria/Vaxzevria  +Spikevax | 3743 (2·0) | 274 (0·9) | **3.05**  **(2.70⎼3.43)** | 0 (0·0) |  |
| *VV/VV+VV* | 17 (0·0) | 4 (0·0) | **10.46**  **(2.85⎼26.78)** | 0 (0·0) |  |
| Janssen+Janssen | 10 (0·0) | 3 (0·0) | **15.75**  **(3.25⎼46.02)** | 0 (0·0) |  |
| Vaxzevria/Vaxzevria  +Janssen | 2 (0·0) | 0 (0·0) |  | 0 (0·0) |  |
| Vaxzevria/Vaxzevria  +Vaxzevria | 5 (0·0) | 1 (0·0) | **7.02**  **(0.18⎼39.1)** | 0 (0·0) |  |
| *mRNA/VV or VV/mRNA + mRNA* | 121 (0·1) | 8 (0·0) | **3.27**  **(1.41⎼6.44)** | 0 (0·0) |  |
| Comirnaty/Janssen  +Comirnaty | 1 (0·0) | 0 (0·0) |  | 0 (0·0) |  |
| Comirnaty/Janssen  +Spikevax | 1 (0·0) | 0 (0·0) |  | 0 (0·0) |  |
| Comirnaty/Vaxzevria  +Comirnaty | 8 (0·0) | 0 (0·0) |  | 0 (0·0) |  |
| Comirnaty/Vaxzevria  +Spikevax | 2 (0·0) | 0 (0·0) |  | 0 (0·0) |  |
| Spikevax/Janssen  +Spikevax | 1 (0·0) | 0 (0·0) |  | 0 (0·0) |  |
| Spikevax/Vaxzevria  +Comirnaty | 4 (0·0) | 0 (0·0) |  | 0 (0·0) |  |
| Vaxzevria/Comirnaty  +Comirnaty | 78 (0·0) | 5 (0·0) | **3.26**  **(1.06⎼7.62)** | 0 (0·0) |  |
| Vaxzevria/Comirnaty  +Spikevax | 10 (0·0) | 2 (0·0) | **9.42**  **(1.14⎼34.01)** | 0 (0·0) |  |
| Vaxzevria/Spikevax  +Comirnaty | 6 (0·0) | 0 (0·0) |  | 0 (0·0) |  |
| Vaxzevria/Spikevax  +Spikevax | 10 (0·0) | 1 (0·0) | **5.83**  **(0.15⎼32.49)** | 0 (0·0) |  |
| *mRNA/VV or VV/mRNA +VV* | 2 (0·0) | 2 (0·0) | **39.37**  **(4.77⎼142.22)** | 0 (0·0) |  |
| Comirnaty/Vaxzevria  +Vaxzevria | 1 (0·0) | 1 (0·0) | **44.05**  **(1.12⎼245.45)** | 0 (0·0) |  |
| Vaxzevria/Comirnaty  +Vaxzevria | 1 (0·0) | 1 (0·0) | **35.59**  **(0.9⎼198.28)** | 0 (0·0) |  |

*mRNA - mesenger ribonucleic acid vaccine

**VV - viral vector vaccine
